# Supplementary material for: Adipose Tissue Stem Cells (ASCs) and ASC-Derived Extracellular Vesicles Prevent the Development of Experimental Peritoneal Fibrosis
Source: Cells. 2025 Mar 14;14(6):436. doi: 10.3390/cells14060436 (PMC11941392; doi:10.3390/cells14060436)
Supplement: Supplementary file 1 [file cells-14-00436-s001.zip › cells-3262303-supplementary.pdf]

Adipose Tissue Stem Cells (ASCs) and ASC-Derived Extracellular Vesicles Prevent the Development of Experimental Peritoneal Fibrosis

Supplementary Materials

Table S1. Criteria to identify ASCs obtained from the 4th cell passage

Morphology	Marker analysis by flow citometry		Differentiation
Fibroblast-like shape	Positive	Negative	Adipocytes
	CD 29 - 96%	CD45 - 7%	Osteoblasts
	CD 90 - 70%		Chondrocytes
	CD 44 - 89%		
	CD 105 - 65%		

Table S2. Analysis of the quantification, gene expression, and peritoneal function in PM

	Control	PF	PF+ASCs	PF+EVs
**Peritoneal Thickness** (μm)	26 ± 1	67 ± 15*	21 ± 3*	24 ± 6#
**α-SMA** (%)	0.1 ± 0.1	1.7 ± 0.6*	0.2 ± 0.2#	0.2 ± 0.1#
**Fibronectin mRNA** (fold change)	1 ± 1	11 ± 1*	1 ± 1#	1 ± 1#
**Collagen III mRNA** (fold change)	1 ± 1	5 ± 1*	4 ± 1*	1 ± 1#§
**TGF-β1 RNA** (fold change)	1 ± 1	15 ± 1*	2 ± 1#	1 ± 1#
**FSP-1 RNA** (fold change)	1 ± 1	17 ± 1*	1 ± 1#	1 ± 1#
**Smad3 mRNA** (fold change)	1 ± 1	7 ± 1*	1 ± 1#	1 ± 1#
**Smad7 mRNA** (fold change)	1 ± 1	2 ± 1	14 ± 2*	11 ± 1*#
**CD68 ED1+** (cells/mm2)	106 ± 35	702 ± 90*	263 ± 58#	148 ± 16#
**CD43** (cells/mm2)	3 ± 1	87 ± 31*	6 ± 3#	11 ± 4#
**M1 macrophage** (%)	-	64 ± 1	54 ± 1#	54 ± 1#
**M2 macrophage** (%)	-	36 ± 1	46 ± 1#	46 ± 1#
**IL-1β RNA** (fold change)	1 ± 1	13 ± 1*	3 ± 2#	3 ± 1#
**TNF-α RNA** (fold change)	1 ± 1	12 ± 1*	1 ± 1#	1 ± 1#
**IL-6 RNA** (fold change)	1 ± 1	26 ± 12*	5 ± 2#	11 ± 7
**Isolectin-B4** (vessels/mm^2)	0.0 ± 0.0	7.8 ± 1.4*	2.3 ± 1.0#	2.4 ± 1.0#
**VEGF RNA** (fold change)	1 ± 1	33 ± 2*	9 ± 1#	4 ± 1#
**Ultrafiltration** (mL)	13 ± 1	3 ± 1*	5 ± 1*	6 ± 1*#
**MTG** (g/KgBW)	889 ± 19	1299 ± 54*	1218 ± 27*	1309 ± 36*

Table S3. Primers used for real-time PCR experiments

TARGET GENE	PRIMERS
**18 S**	Forward 5' AGGAGTACGATGAGTCCGGCCC 3'<br>Reverse 5' GCAGCTCAGTAACAGTCCGCCT 3'
**Pro-Collagen III**	Forward 5' GCAATACCAGGAGCACCATT 3'<br>Reverse 5' AGCTGGTGCTAAGGGTGAAG 3'
**Fibronectin**	Forward 5' TGACCCAGACTTACGGTGGCA 3'<br>Reverse 5' GGAGTAGAAGGTCCTACCGTTGTAGTG 3'
**FPS-1**	Forward 5' GGCAACGAGGGTGACAAGTT 3'<br>Reverse 5' CCCTGGTCAGTAGTCCCTTGA 3'
**Smad 3**	Forward 5' TCAACGGAACTTGGGAATGAG 3'<br>Reverse 5' GTAGTGCGGAGCTCTCCTTCA 3'
**Smad7**	Forward 5' CCTGGCCGGTGTAAATGTCT 3'<br>Reverse 5' GCGGATCCCTTGGAAAGG 3'
**TGF- β**	Forward 5' CAACCCGGGTGCTTCCGCAT 3'<br>Reverse 5' TGCTCCACCTTGGGCTTGCG 3'
**TNF-α**	Forward 5' TGGCCCAGACCCTCACACTCA 3'<br>Reverse 5' GGCTCAGCCACTCCAGCTGC 3'
**VEGF**	Forward 5' ACTGTGAGCCTTGTTCAGAGCGG 3'<br>Reverse 5' TCAAGCTGCCTCGCCTTGCA 3'

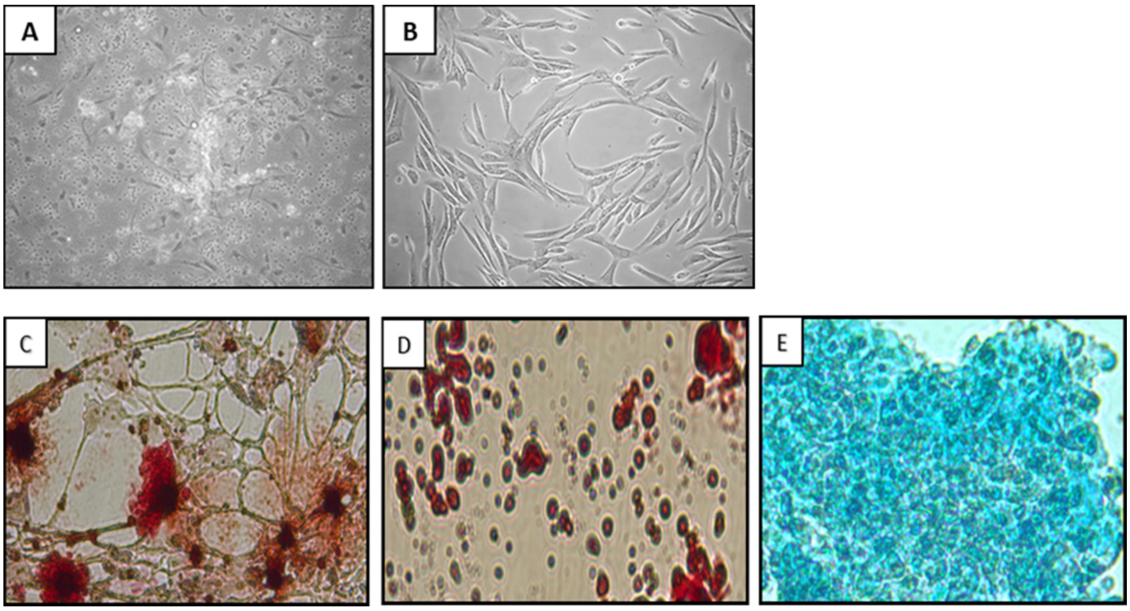

Figure S1. ASC culture and characterization. ASC spindle cells in culture in passages **(A)** P0 and **(B)** P4, 400×. Differentiation of ASCs in **(C)** osteoblast-like cells; **(D)** adipocytes; and **(E)** chondrocytes and osteoblast-like cells.

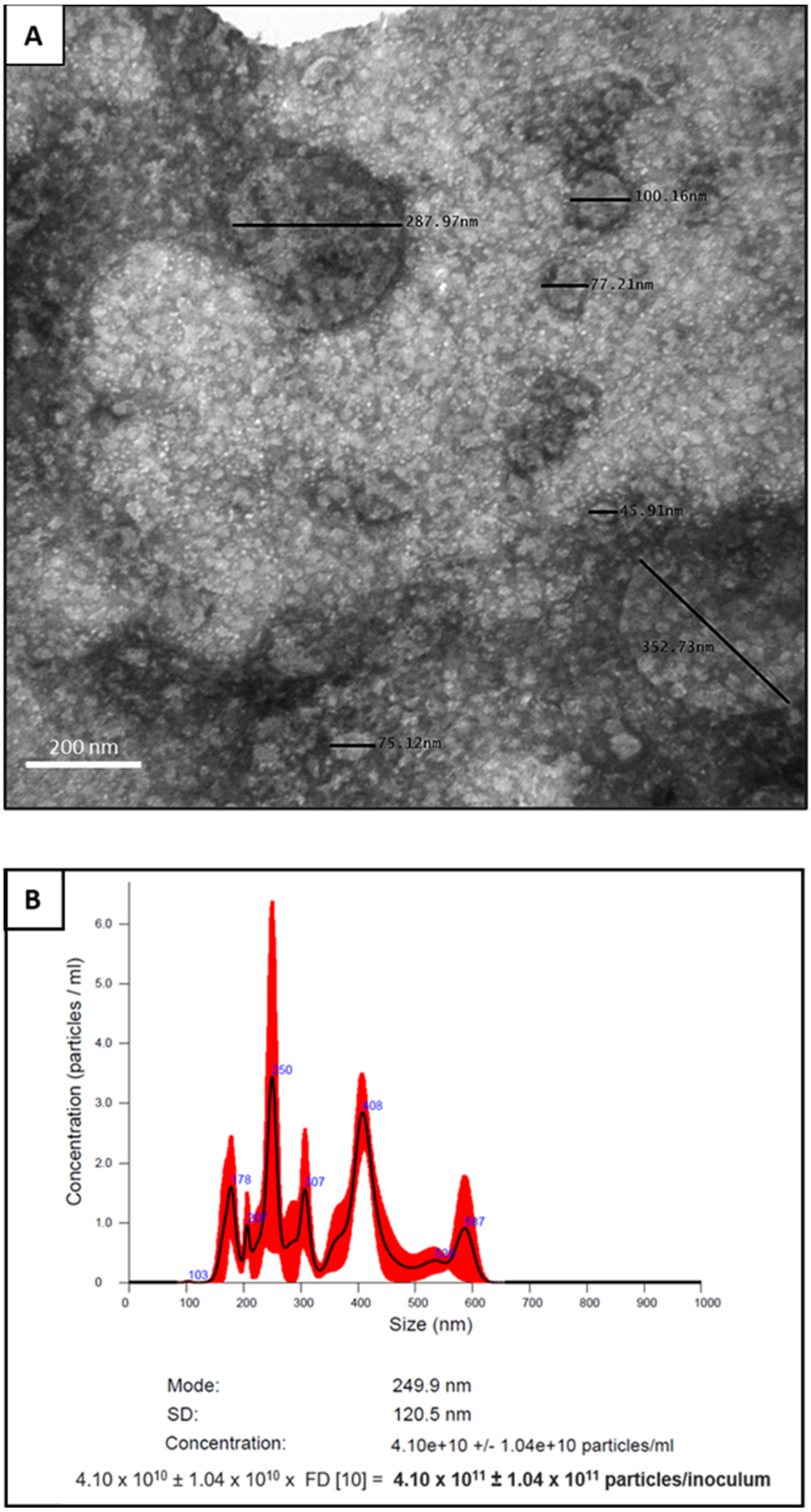

Figure S2. Characterization of EVs derived from ASCs. **(A)** EVs were morphologically characterized using a JEM 1011 JEOL transmission electron microscope, at 80 kV (Carl Zeiss NTS, Thuringia, Germany). **(B)** The number and size distribution of EVs were analyzed in a NANOSIGHT 3 nanoparticle tracking analysis (NTA) device (NanoSight Ltd). After correction for the dilution factor, we found that each EV inoculum comprised approximately 4 × 10^{11} particles, varying in size between 50 and 600 µm; particles with an approximate diameter of 250 µm were predominant.

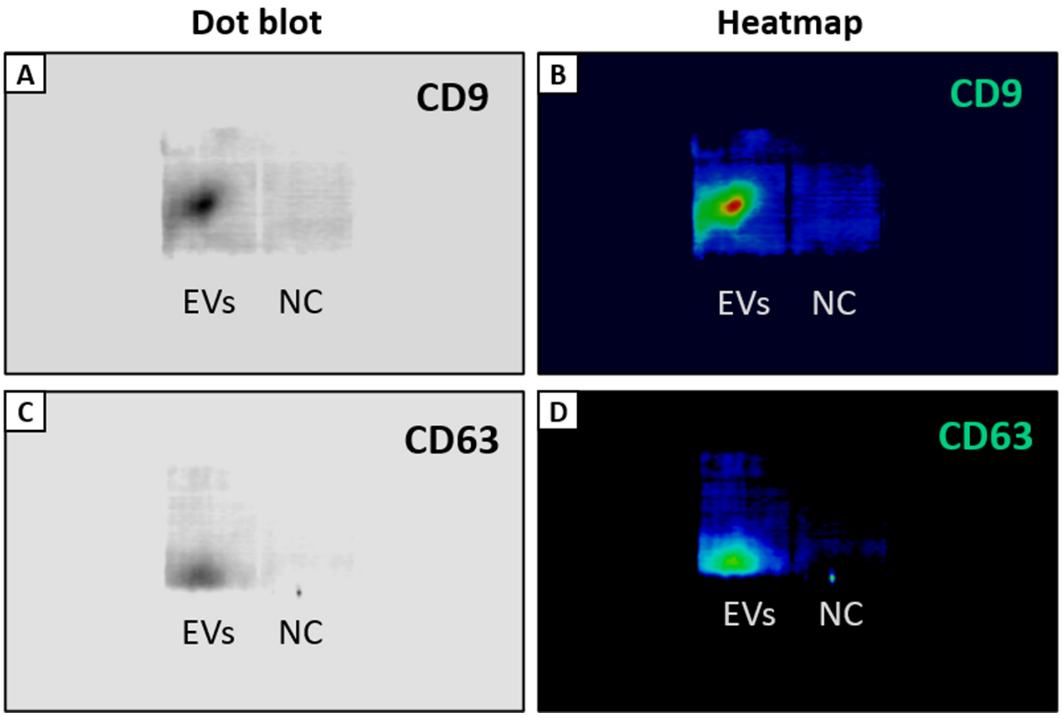

Figure S3. EVs were characterized by their positivity for tetraspanins CD9 and CD63. Dot blot analyses were performed in spots of 10 µL of EV solution, containing approximately 10 µg of total protein each. Positivity of EVs for CD9 **(A)** and CD63 **(B)** could be seen in ECL-developed nitrocellulose membranes and analyzed by the heatmap tool of a UVITEC Cambridge image acquisition system (**C**,**D**). Negative controls (NC) were performed by spotting 10 µL of bovine serum albumin (BSA) solution, containing 10 µg of BSA, in the same nitrocellulose membranes.

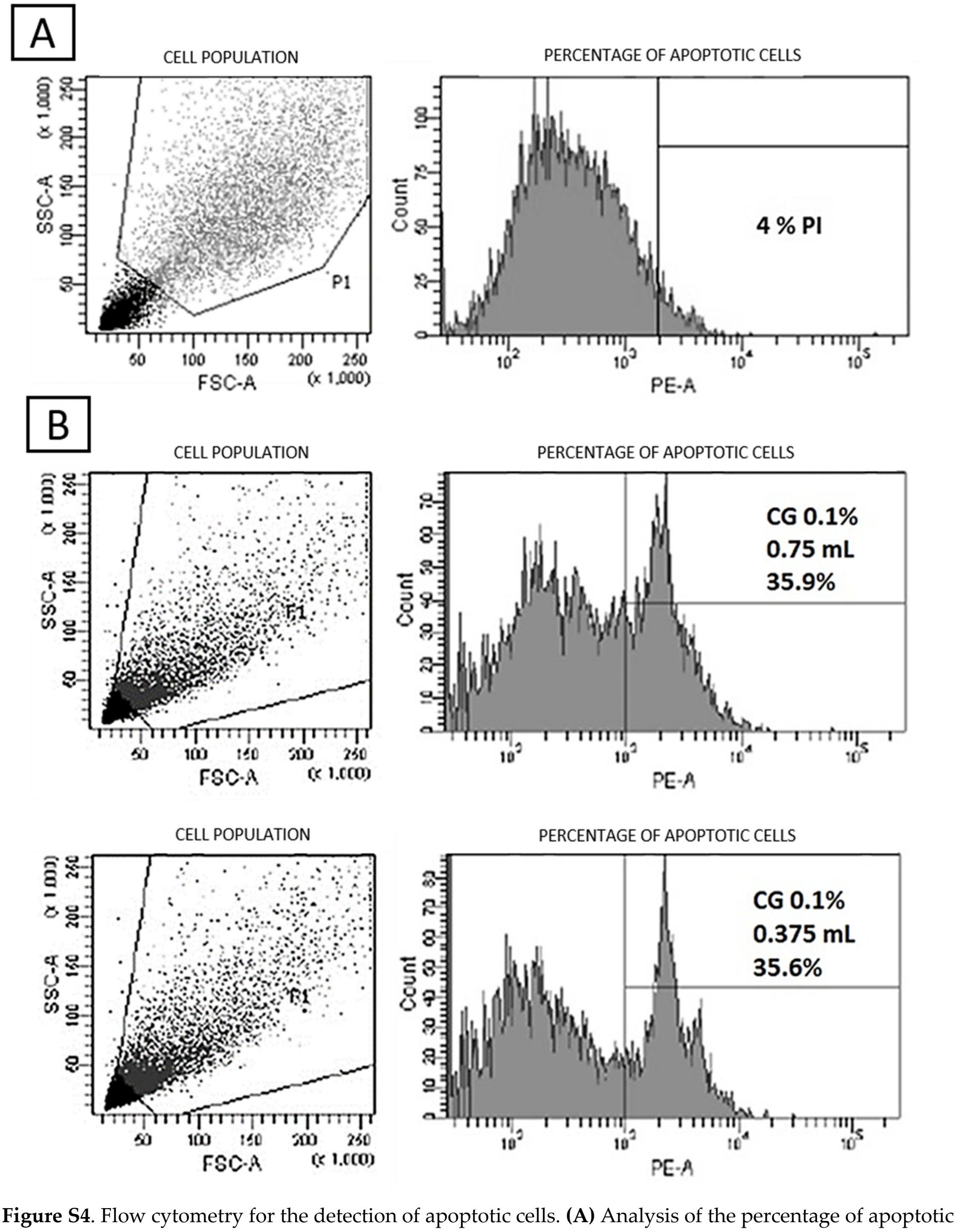

Figure S4. Flow cytometry for the detection of apoptotic cells. **(A)** Analysis of the percentage of apoptotic ASCs after culture for 24 h without FBS in the medium. **(B)** Analysis of the percentage of apoptotic ASCs after culture for 24 h with 0.1% CG.
